# Discriminative-Region Multi-Label Classification of Ultra-Widefield Fundus Images

**DOI:** 10.3390/bioengineering10091048

**Published:** 2023-09-06

**Authors:** Van-Nguyen Pham, Duc-Tai Le, Junghyun Bum, Seong Ho Kim, Su Jeong Song, Hyunseung Choo

**Affiliations:** 1 Department of Electrical and Computer Engineering, Sungkyunkwan University, Suwon 16419, Republic of Korea; nguyenpv195@g.skku.edu; 2 College of Computing and Informatics, Sungkyunkwan University, Suwon 16419, Republic of Korea; ldtai@skku.edu; 3 Sungkyun AI Research Institute, Sungkyunkwan University, Suwon 16419, Republic of Korea; bumjh@skku.edu; 4 Department of Ophthalmology, Kangbuk Samsung Hospital, School of Medicine, Sungkyunkwan University, Seoul 03181, Republic of Korea; n09072@gmail.com; 5 Biomedical Institute for Convergence, Sungkyunkwan University, Suwon 16419, Republic of Korea; 6 Department of Superintelligence Engineering, Sungkyunkwan University, Suwon 16419, Republic of Korea

**Keywords:** automated disease classification, deep learning, multi-label classification, ocular diseases, ophthalmology, ultra wide-field fundus images

## Abstract

Ultra-widefield fundus image (UFI) has become a crucial tool for ophthalmologists in diagnosing ocular diseases because of its ability to capture a wide field of the retina. Nevertheless, detecting and classifying multiple diseases within this imaging modality continues to pose a significant challenge for ophthalmologists. An automated disease classification system for UFI can support ophthalmologists in making faster and more precise diagnoses. However, existing works for UFI classification often focus on a single disease or assume each image only contains one disease when tackling multi-disease issues. Furthermore, the distinctive characteristics of each disease are typically not utilized to improve the performance of the classification systems. To address these limitations, we propose a novel approach that leverages disease-specific regions of interest for the multi-label classification of UFI. Our method uses three regions, including the optic disc area, the macula area, and the entire UFI, which serve as the most informative regions for diagnosing one or multiple ocular diseases. Experimental results on a dataset comprising 5930 UFIs with six common ocular diseases showcase that our proposed approach attains exceptional performance, with the area under the receiver operating characteristic curve scores for each class spanning from 95.07% to 99.14%. These results not only surpass existing state-of-the-art methods but also exhibit significant enhancements, with improvements of up to 5.29%. These results demonstrate the potential of our method to provide ophthalmologists with valuable information for early and accurate diagnosis of ocular diseases, ultimately leading to improved patient outcomes.

## 1. Introduction

Ultra-widefield fundus image (UFI) is an advanced image modality that has revolutionized the diagnosis and treatment of ocular diseases. Unlike conventional fundus photographs (FP), which capture only a small portion of the retina with a field of view from 30 to 40 degrees, UFI provides an ultra-wide view of the retina up to 300 degrees. This extended view enables the identification of peripheral diseases that may not be visible in conventional FP [1,2]. However, the low resolution of macular area, frequent artifacts, and distortion of pseudo color images hinder the accurate interpretation of UFI [3]. As a result, there is an increasing need for a precise automatic disease classification system in UFI to ensure rapid and accurate diagnosis of multiple macular and peripheral ocular diseases, which is crucial for vision preservation.

Multi-label classification of UFI faces several challenges due to the complexity of ocular diseases and the vast amount of information captured by UFI images. First, UFI images contain a large amount of noise and artifacts, which obscure important features and make the identification of diseases difficult. Second, multiple diseases can occur in one eye, and the lesions can overlap, making it challenging to accurately classify and localize each disease. Moreover, the manifestations of a single disease can vary drastically among patients, as it may present diverse features and patterns, thereby adding another layer of complexity to the classification process. In addition, the scarcity of labeled UFI data poses a challenge, as manual annotation is a time-consuming process that requires significant expertise. Addressing these challenges is essential to develop accurate and efficient multi-label classification methods for UFI, which can significantly improve the diagnosis and management of ocular diseases.

Despite significant progress in the use of UFI for diagnosing and managing ocular diseases, current classification methods still have limitations. Many existing studies focus only on single diseases, such as diabetic retinopathy [4,5,6,7], glaucoma [8], or retinal detachment [9,10,11]. On the other hand, some studies have made attempts at classifying multiple diseases but limit their data to images containing only a single disease [12,13], making it less practical for real-world applications. Although some works have tackled the multi-label classification problem [14,15] and achieved impressive results, they fail to leverage the unique features of each disease to improve classification performance. Therefore, it is crucial to develop a novel strategy that considers the distinct characteristics of each disease to overcome the challenges of multi-label classification in UFI. By incorporating disease-specific information, this approach has the potential to provide a more comprehensive and accurate classification of ocular diseases.

**Figure 1:**
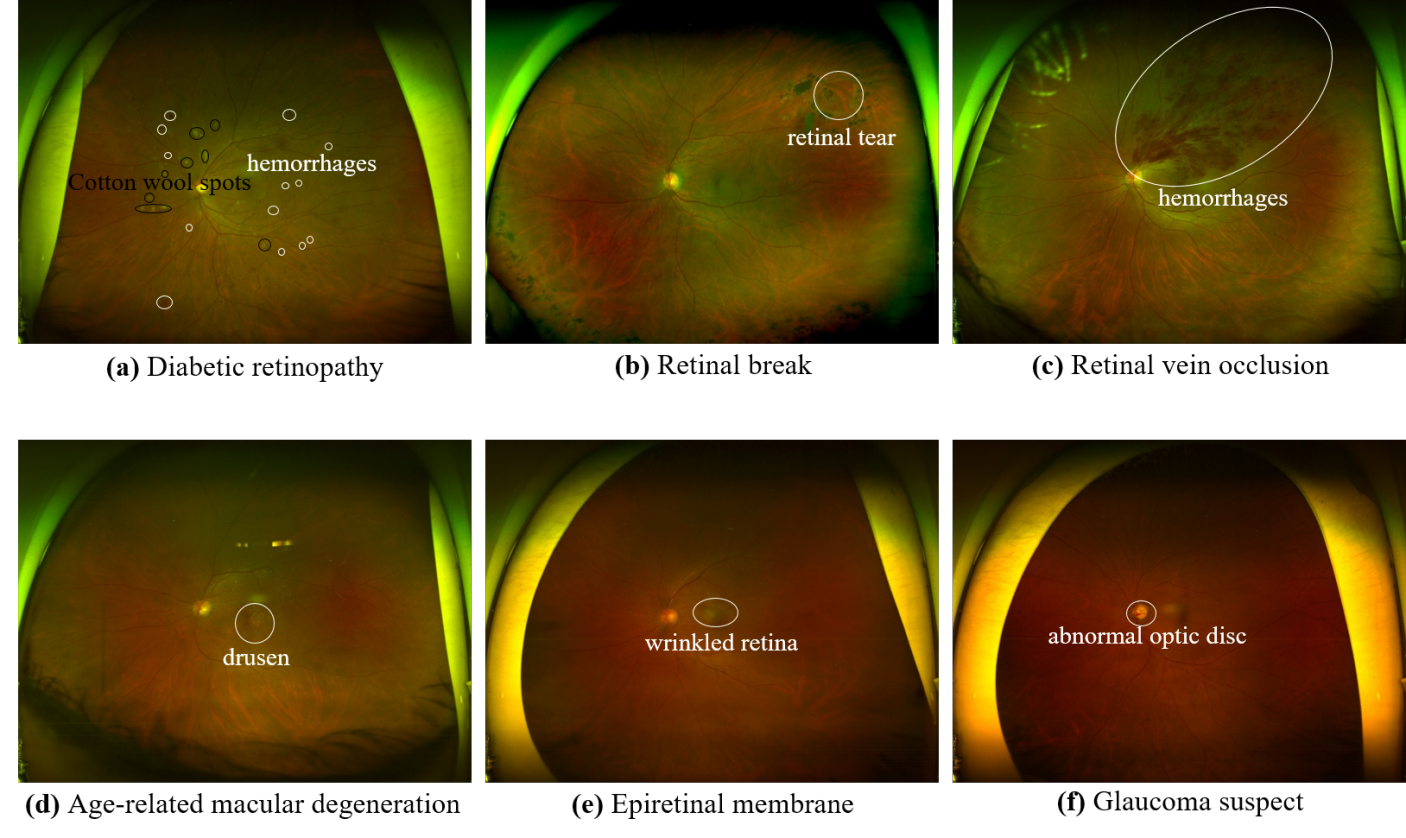
Lesions’ distribution of each disease.

In this study, we present a novel method to enhance the efficiency of UFI classification by utilizing disease-specific knowledge. Initially, we construct a dataset encompassing six distinct ocular diseases: diabetic retinopathy (DR), retinal break (RB), retinal vein occlusion (RVO), epiretinal membrane (ERM), age-related macular degeneration (AMD), and glaucoma suspect (GS). Based on the spatial distribution of typical lesions associated with each disease, as depicted in Figure 1, we observe that DR, RB, and RVO lesions distribute across the UFI, while AMD and ERM predominantly manifest in the macular region, and GS-related anomalies mainly emerge near the optic disc. The main idea of our innovative approach is to utilize the entire UFI for DR, RB, and RVO classification, focus on the macular region for ERM and AMD diagnosis, and target the optic disc vicinity for GS detection. By employing Faster R-CNN, we accurately localize the optic disc and macula which are used to generate two regions of interest (ROIs): the optic disc area and the macular area. Subsequently, these ROIs, alongside the entire UFI, undergo processing by deep learning-based classification models to detect various ocular diseases. Our proposed method outperforms existing works across various metrics on a dataset comprising 5930 UFIs. In particular, our method achieves an AUC score and accuracy up to 99.14% and 95.79%, respectively. These results highlight the potential of our method to provide ophthalmologists with valuable information for effective diagnosis and management of ocular diseases, ultimately improving patient outcomes.

The rest of this paper is organized as follows: Section 2 provides a review of the related work in the field of ultra-widefield fundus image classification. Section 3 presents a comprehensive description of the materials and the proposed method, including the utilization of disease-specific regions of interest. In Section 4, we outline the experimental setup as well as evaluation metrics and report the results obtained from our experiments. Finally, Section 5 concludes the paper by summarizing the key findings and discussing the implications of our proposed method for ocular disease diagnosis.

## 2. Related Work

Recently, deep learning has significantly improved the interpretation of fundus images, including tasks like vessel segmentation [16,17], optic disc and fovea (the center of the macula) localization [18,19], and image registration [20,21]. For vessel segmentation, researchers in [16] noticed that existing UNet-based models [22] can lose information due to multiple pooling steps and insufficient processing of local context features. To address this, they introduced a pooling fusion block that combines max pooling and average pooling layers to merge information. Additionally, an attention fusion block was created to better capture features at different scales. For optic disc and fovea localization, a two-stage method in [18] integrates deep learning with a mathematical model. They first locate the optic disc using Faster RCNN [23], and then use its position to find the fovea within a ring-shaped area. In one paper [20], the authors proposed an unsupervised approach for retina fundus registration by combining two deep learning-based networks. A U-shaped fully convolutional neural network [24] and a spatial-transformer-type network [25] work together: the former finds matching points in fundus images, and the latter uses these points for geometric bilinear interpolation. This method achieves registration without needing prelabeled data or synthetic training techniques. Once fully trained, this integrated approach can perform one-shot registrations with a given pair of fundus images.

Over the years, classic machine learning methods such as Naive Bayesian (NB), random forest (RF), support vector machine (SVM), and *k*-nearest neighbor (KNN) have proven to be robust tools in the detection of ocular diseases, outperforming traditional handcrafted methods. In one paper [26], researchers examined the performance of four machine learning algorithms, C5.0, RF, SVM, and KNN, in detecting glaucoma based on the thickness of the retinal nerve fiber layer and visual field. The study found that RF offered the best performance, while the others delivered comparable accuracy. In another work [27], a machine learning-based method was proposed to detect microaneurysms (MA), which is the earliest symptom of DR. The method involved segmenting and removing blood vessels from the images to reduce extraneous information. Subsequently, MA candidate regions were identified using shape characteristics and connected components analysis. Each candidate region was then divided into patches, and features were extracted from each patch for classification as MA or non-MA. Three algorithms were applied for this final classification: NB, KNN, and SVM. The experiment results suggested that NB yielded the best performance. In another paper [28], for AMD detection, the authors proposed a method consisting of two main phases: image processing and pattern classification. In the image processing phase, standard image processing techniques and mathematical morphology operations were applied to extract the AMD pattern. In the second phase, the generated pattern from the fundus image was fed into an SVM classifier, which categorized it into one of two classes: AMD or non-AMD.

Numerous studies have focused on single-disease detection. In one paper [4], the authors investigate DR grading classification using two imaging modalities: UFI and optical coherence tomography angiography (OCTA), as well as their combination. Their study involves two tests: identifying no apparent DR (NDR) or DR, and distinguishing between NDR and proliferative DR. The results demonstrate that for the first test, OCTA alone achieves the best performance, whereas for the second test, UFI alone leads to superior results. This finding suggests that single modalities can outperform combined modalities. The study in [9] evaluates the performance of ResNet50 and InceptionResNetV2 in detecting recurrent retinal detachment, with both models delivering impressive results that exceed those of junior ophthalmologists. For multi-disease single-label classification, the authors of [29] utilize DenseNet121 to detect three diseases: DR, sickle cell retinopathy, and RVO. Meanwhile, in [12], six preprocessing techniques and a model ensemble strategy are employed for a screening system targeting three diseases: DR, retinal detachment, and myopia. For multi-label classification, the authors of [30] implement separate classifiers for each disease, while the authors of [31] apply histogram equalization to both grayscale and original images, with the final output determined based on the classification results of the two equalized images. Moreover, in [14], a multi-label classification system based on a customized ResNet34 is introduced. To enhance performance, various techniques such as data augmentation, model exponential moving average, and mix-up training strategy are employed.

## 3. Materials and Methods

### 3.1. Dataset and Disease Description

**Figure 2:**
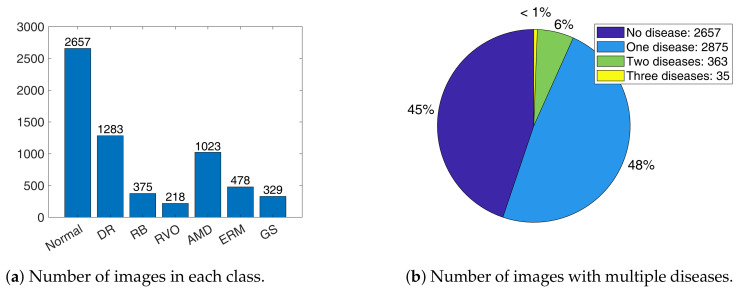
Dataset statistics.

**Figure 3:**
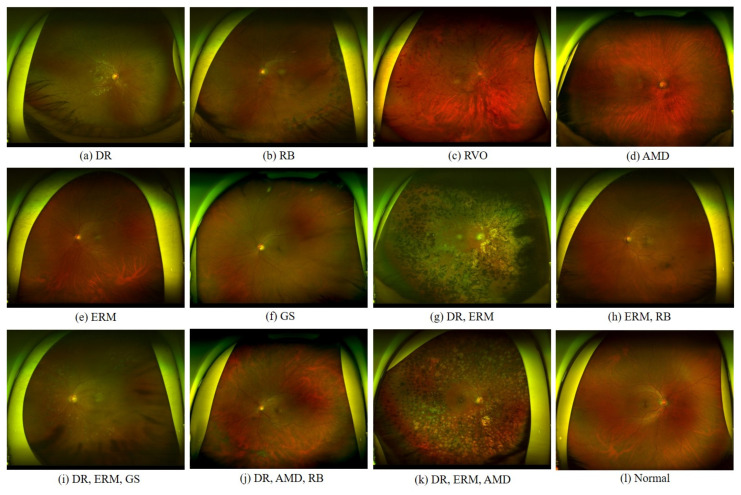
Samples of images with one disease: (**a**–**f**), two diseases: (**g**,**h**), three diseases: (**i**–**k**), and normal: (**l**).

In this study, we utilized a UFI dataset comprising patients diagnosed with various ocular diseases. The dataset contains 5930 UFIs collected from 2 January 2006 to 31 December 2019, from patients who received eye examinations at the Department of Ophthalmology, Kangbuk Samsung Hospital. This study was approved by the Institutional Review Board of Kangbuk Samsung Hospital on 4 February 2020 (approval number: KBSMC 2020-01-031). The images were annotated by experienced ophthalmologists to identify the presence or absence of six common ocular diseases: diabetic retinopathy (DR), retinal breaks (RB), retinal vein occlusion (RVO), epiretinal membrane (ERM), age-related macular degeneration (AMD), and suspected glaucoma (GS). Figure 2a describes the statistics of the dataset, while Figure 3 shows images with different numbers of diseases. This dataset serves as a valuable resource for developing and evaluating multi-label classification models for UFIs. The six diseases in the dataset are described below:

- DR is a diabetes complication that affects blood vessels in the retina. DR is marked by microaneurysms, hemorrhages, hard exudates, cotton wool spots, neovascularization, and vitreous hemorrhage. These lesions typically appear in the retina’s periphery.
- RB is a type of retinal disorder involving the detachment or separation of the retina from the underlying tissue. Retinal breaks are characterized by a retinal tear or hole. These lesions can occur anywhere on the UFI.
- RVO is a blockage of retinal veins, causing blood and fluid accumulation in the retina. RVO is marked by retinal hemorrhages, cotton wool spots, macular edema, and neovascularization. These lesions generally appear in the central and mid-peripheral retina on UFI.
- ERM is a condition where a thin tissue layer grows on the retina’s surface, causing visual distortion. ERM is characterized by a wrinkled or folded retina, cystic spaces, and macula distortion. These lesions typically appear in the macular region on UFI.
- AMD is a condition in which the macula, responsible for central vision, deteriorates over time. AMD is marked by drusen, pigmentary changes, geographic atrophy, and neovascularization. These lesions generally appear in the macular region on UFI.
- GS refers to situations that might develop glaucoma. Glaucoma is a group of eye conditions that damage the optic nerve and can lead to blindness. Some of the findings on UFI that could indicate a GS include optic disc changes and retinal nerve fiber layer defects. These findings occur in the area surrounding the optic disc.

### 3.2. Proposed Method

**Figure 4:**
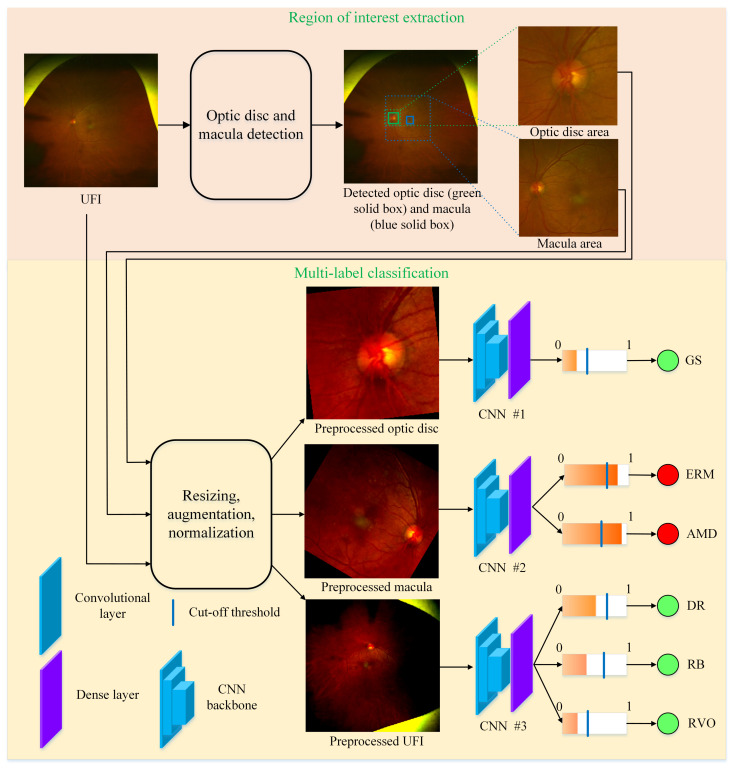
Overall process of the proposed method. Each output from CNNs represents the probability of containing a specific disease. If this value is greater than the cut-off threshold, the image is predicted as containing disease, otherwise, non-disease.

From the characteristics of the diseases, we see that the lesions of DR, RB, and RVO scatter on the entire UFI. While the lesions of ERM and AMD typically appear surrounding the macula area, the diagnosis of GS is mostly based on the optic disc. Based on this observation, our idea is to extract three ROIs: the entire UFI, the macula area, and the optic disc area; each ROI is used for the diagnosis of its corresponding disease(s). The overall process of our method is illustrated in Figure 4. Our method includes two stages: region of interest extraction and multi-label classification.

#### 3.2.1. Region of Interest Extraction

**Figure 5:**
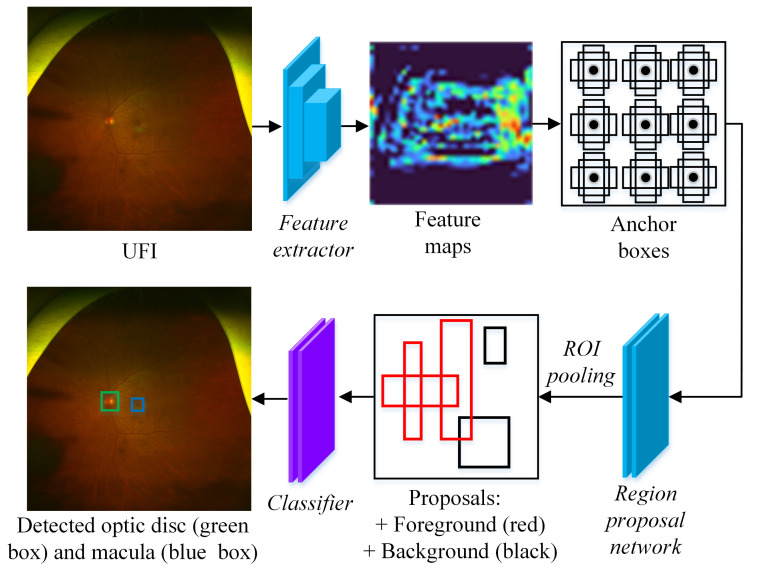
The process of detecting the optic disc and macula.

Our approach begins with the detection of the optic disc and macula, as illustrated in Figure 5. These biomarkers are then used to identify relevant areas within the UFI. To locate the optic disc and macula in the UFI, we employ the Faster RCNN detector [23], a two-stage object detection model known for its accuracy and efficiency. In the first stage, the UFI is passed through a feature extractor consisting of 13 convolutional layers from VGG16 [32] network. Each layer computes a transformation of the input image, which results in a set of feature maps. For instance, the early layers of the feature extractor might capture basic features like edges and textures, while deeper layers might capture more complex patterns or shapes that are indicative of specific biomarkers or lesions. For each spatial location in the feature map, a set of anchor boxes is generated. Anchor boxes are not manually annotated in the training data. Instead, they are predefined geometric shapes (rectangles) that are designed to cover a wide range of object sizes and aspect ratios [23]. In experiments, for each spatial location on the feature map, nine predefined anchor boxes are generated. During the training, the region proposal network refines the positions and assigns the objectness scores of those boxes. The refined boxes are called proposals. Based on the objectness score, each proposal is classified into either foreground (containing objects) or background (not containing objects). To reduce computational complexity and improve the detection process, the proposals are passed into the ROI pooling step. Any proposals crossing image boundaries are removed. In addition, proposals overlapping others with an Intersection over a Union score greater than 0.7 are eliminated. This step is crucial for reducing redundancy and retaining only the most relevant proposals. In the second stage, the remaining 256 proposals are sent to a classifier for accurately predicting the categories (optic disc, macula, or background) and refining the bounding box coordinates for each object. If multiple boxes are predicted as the optic disc or macula, the one with the highest probability is chosen as the final result, since there is exactly one optic disc and one macula in each UFI.

**Figure 6:**
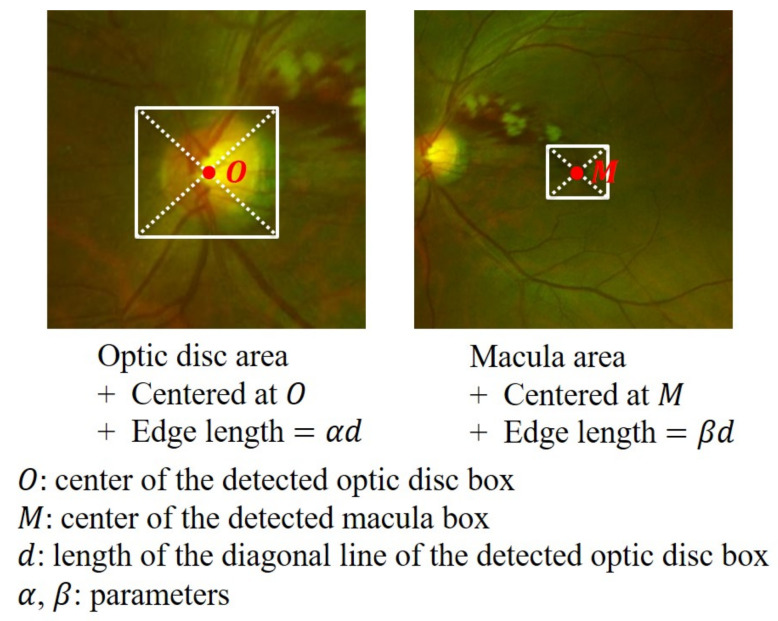
Optic disc and macula areas.

Following the detection of the optic disc and macula, we proceed to extract two ROIs based on their respective locations. Our objective is to maintain a consistent area covered by the ROIs across different images. To accomplish this, we aim to associate the ROIs’ area with a fixed size related to either the optic disc or the macula. In UFIs, the macula’s boundary is often unclear, resulting in a wide variation in the detected macula size across different images. Conversely, the optic disc is more distinct and easily detected, leading to a more consistent size throughout various images. As such, we choose to link the size of the ROIs to the size of the detected optic disc. The two ROIs are illustrated in Figure 6, where both the optic disc area and macula area are represented as squares. The optic disc area is centered at the detected optic disc box’s center, denoted as *O*, and has an edge length of αd. Similarly, the macula area is centered at the detected macula box’s center, represented as *M*, and has an edge length of βd. Here, *d* refers to the length of the diagonal of the detected optic disc box, while α and β are adjustable parameters that determine the edge length. By linking the ROIs’ size to the detected optic disc and maintaining a consistent area across images, we can effectively focus on the most relevant regions for subsequent classification tasks, ultimately enhancing the overall performance of our method.

#### 3.2.2. Multi-Label Classification

The second stage of our method starts with data preprocessing which is essential in preparing images for deep learning models, as it ensures consistency and diversity within the dataset. First, all images are resized to a uniform size, providing the model with consistent input across the entire dataset. This step streamlines the training process and enhances its efficiency. Next, online data augmentation techniques, such as random horizontal flip and random rotation, are employed to increase the dataset’s diversity. The random horizontal flip is applied with a probability of 0.5. For a dataset with *N* images, on average, N/2 images would be flipped during any single epoch. For the random rotation, each image in the dataset is rotated randomly by a degree between −180 and +180 for every batch during training. Given the continuous and random nature of this augmentation, it results in virtually endless possible variations for each image over multiple epochs. These online data augmentation techniques improve the model’s ability to generalize and recognize patterns in previously unseen data. By incorporating minor variations in the images, the model becomes more robust and less susceptible to overfitting. Following augmentation, the images undergo normalization, a process that adjusts pixel values to a standard scale. Normalization not only accelerates the training process but also fosters better convergence and overall model performance. In summary, the data preprocessing stage serves to create a well-prepared and diverse dataset, empowering the classification model to perform more effectively and accurately in identifying various ocular diseases in UFI.

The multi-label classification process in this study involves utilizing three distinct regions of interest (ROIs): optic disc area, macula area, and the entire UFI. Each ROI is processed through a convolutional neural network (CNN) to identify one or more diseases. Particularly, the optic disc area is used to detect GS, while the macula area is employed to identify ERM and AMD. In contrast, the entire UFI is utilized to detect DR, RB, and RVO. To effectively train the CNNs, appropriate loss functions are employed. For the CNN responsible for detecting GS, the CrossEntropyLoss function provided by Pytorch [33] is used. This loss function is suitable for single disease classification tasks, such as detecting GS in the optic disc area. On the other hand, the BCEWithLogitsLoss function, also provided by Pytorch, is employed for the remaining two CNNs, which handle the multi-label classification of the macula area and the entire UFI. This loss function is designed to handle multi-label classification tasks, making it suitable for detecting multiple diseases in the macula area and the entire UFI. Through this multi-label classification process, the proposed method efficiently identifies various ocular diseases in different regions of UFI, contributing to more accurate diagnoses and improved patient outcomes.

## 4. Performance Evaluation

### 4.1. Implementation Details and Evaluation Metrics

In our study, we carefully established implementation settings to ensure optimal performance and reproducibility. For the task of optic disc and macula detection, we annotate the locations of optic disc and macula in 1204 labeled UFIs. The training procedure of Faster-RCNN is based on the TensorFlow object detection API. In the classification task, each class is divided into training and validation sets with an approximate ratio of 9:1. Each CNN backbone is initialized with weights learned from the large-scale ImageNet dataset. All CNNs are trained using the Adam optimizer and a batch size of 8. The learning rate is scheduled to decrease by a factor of 0.5 every 10 epochs. CNNs #1 and #2 are initialized with a learning rate of 1 × 10^−3^, while CNN #3 starts with a learning rate of 1 × 10^−4^. Each CNN is trained for a total of 60 epochs, and the model exhibiting the highest average validation AUC for all classes is selected as the best model.

For each class, we consider four metrics, including AUC, accuracy, sensitivity, and specificity. To assess the overall performance across all classes, we employ micro-average (μ-) accuracy, micro-average sensitivity, and micro-average specificity. These micro-average metrics are beneficial for imbalanced datasets, as they take into account the number of images in each class, with classes having more images exerting a greater influence on the final result. AUC is a popular evaluation metric in classification tasks. It assesses the model’s proficiency in accurately ranking the predicted probabilities of positive samples higher than those of negative samples. By considering the overall distribution of probabilities, AUC captures the model’s ability to distinguish between positive and negative samples. A higher AUC value indicates a better model, with a perfect classifier achieving an AUC score of 1. We use AUC as the main metric for performance evaluation.

Accuracy is the proportion of correct predictions among the total number of cases examined. It is a commonly used metric to assess the overall performance of a classification model. Sensitivity measures the proportion of true positive cases among the actual positive cases. It is an essential metric to evaluate the model’s ability to correctly identify positive cases or detect the presence of a specific condition. Specificity measures the proportion of true negative cases among the actual negative cases. It evaluates the model’s ability to correctly identify negative cases or rule out the presence of a specific condition. Micro-average accuracy is calculated by summing up the true positives, true negatives, false positives, and false negatives across all classes and then computing the accuracy. Micro-average sensitivity is obtained by summing up the true positives and false negatives across all classes and then computing the sensitivity. Micro-average specificity is calculated by summing up the true negatives and false positives across all classes and then computing the specificity. Let TP, TN, FP, and FN be the number of true positives, true negatives, false positives, and false negatives, respectively, and *n* be the number of classes in the dataset, equations for the above metrics are:

$$accuracy=\frac{TP+TN}{TP+TN+FP+FN}\;\mu \text{-}accuracy=\frac{\sum_{i=0}^{n}\left(TP_{i}+TN_{i}\right)}{\sum_{i=0}^{n}\left(TP_{i}+TN_{i}+FP_{i}+FN_{i}\right)}$$

$$sensitivity=\frac{TP}{TP+FN}\;\mu \text{-}sensitivity=\frac{\sum_{i=0}^{n}TP_{i}}{\sum_{i=0}^{n}\left(TP_{i}+FN_{i}\right)}$$

$$specificity=\frac{TN}{TN+FP}\;\mu \text{-}specificity=\frac{\sum_{i=0}^{n}TN_{i}}{\sum_{i=0}^{n}\left(TN_{i}+FP_{i}\right)}$$

In our study, we adopt a one-vs-rest evaluation for each disease. For each specific disease, we categorize it as the positive class, while classifying all other diseases, including the normal state, as the negative class. As a result, the reported performance of each class specifically highlights its proficiency in differentiating a particular disease (or group of diseases) from the remaining classes. For example, the performance of GS measures the model’s competence in distinguishing GS from all other conditions, including both the normal state and other diseases. To guarantee robustness in our findings, we conducted the experiments five times and reported the results within a 95% confidence interval (mean±variation). This interval suggests that, if the experiment were repeated 100 times, the outcome would fall within the range of [mean−variation,mean+variation] in 95 out of those 100 times.

### 4.2. Experiment Results

#### 4.2.1. Comparison with Existing Works

In this section, we compare our method’s performance with the studies by Lee et al. [30], Wang et al. [31], and Zhang et al. [12]. We previously reviewed these works in Section 2. For our method, we employ EfficientNetB3 for the CNNs #1 and #2, and Xception for CNN #3. The macula area size is set at 6 *d*, while the optic disc area size is set to 3.00 *d*. We will discuss the rationale behind these choices in the following section.

Table 1 presents the AUC scores for each disease, offering a comprehensive evaluation of the proposed method against the three reference methods. The proposed method surpasses the reference methods across all six diseases, showcasing its enhanced diagnostic capabilities. Notably, our method attains significant performance improvements for GS, ERM, DR, and AMD, with AUC score increases of 5.29%, 5.05%, 2.13%, and 2.13%, respectively, compared to the top-performing reference method. This underlines our method’s effectiveness in accurately diagnosing these specific ocular conditions. In addition, our method exhibits better performance for RB and RVO, improving by 0.59% and 0.11% over the highest-scoring reference method. This demonstrates that the proposed method not only excels in detecting GS, ERM, DR, and AMD but also provides competitive results for RB and RVO relative to other approaches. Collectively, these outcomes emphasize the robustness of the proposed classification model in diagnosing a range of ocular diseases, highlighting its potential to contribute to improved patient outcomes.

**Figure 7:**
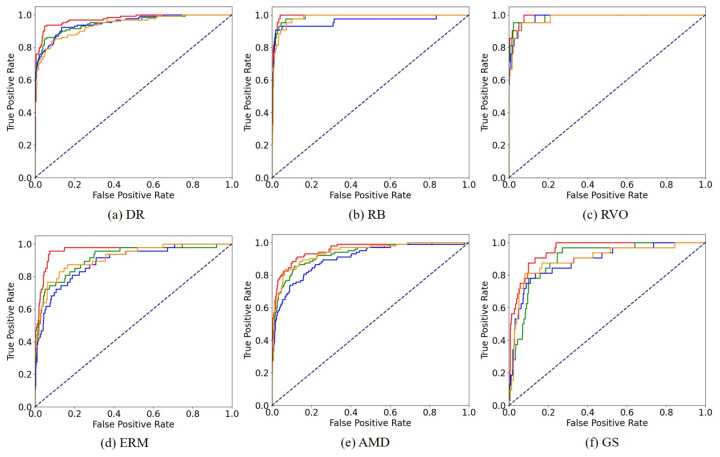
ROC curves for each disease. The curve closer to the top left corner indicates better performance. Red: Proposed, Orange: Lee et al. [30], Green: Wang et al. [31], Blue: Zhang et al. [12], Dashed line: random classifier.

To verify the reliability of our method’s performance and to ensure that the improvements are not solely due to random variation, we conduct significance tests for AUC scores to establish that our advancements over existing works are statistically significant. Table 2 displays the results of these significance tests, represented by *p*-value. In most cases, the improvements of the proposed method are statistically significant compared to the reference methods. Figure 7 provides a visual representation of the AUC scores by illustrating the ROC curves for all methods. A curve closer to the top-left corner indicates superior performance. Our method outshines the reference methods for four diseases (DR, RB, ERM, AMD, and GS) and demonstrates competitive performance for RVO.

The cut-off threshold is a critical component in the classification process, as it determines the point at which a predicted probability from a classification model is considered a positive or negative class label. Although the default threshold is often set at 0.5, this value may not be optimal for all problems or datasets, particularly in imbalanced or complex multi-label situations. The cut-off threshold significantly influences a model’s sensitivity and specificity. Lowering the threshold results in a more aggressive classifier, predicting more positive instances and thus increasing sensitivity at the expense of specificity due to a higher false positive rate. Conversely, raising the threshold makes the classifier more conservative, leading to a higher true negative rate and improved specificity, but at the cost of reduced sensitivity. As both sensitivity and specificity are crucial, we tune the cut-off threshold for the proposed and reference methods to maximize the product (sensitivity × specificity), aiming to balance these two measurements. The threshold for DR, RB, RVO, ERM, AMD, and GS are 0.11, 0.04, 0.02, 0.12, 0.30, and 0.06, respectively.

**Figure 8:**
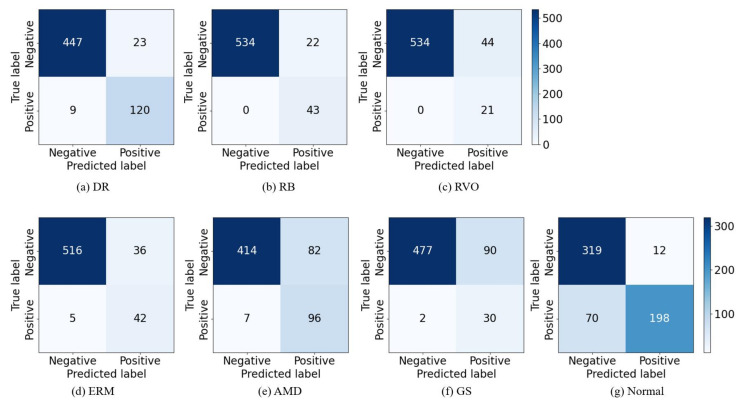
Confusion matrix for each class of our method. For each class, “positive” refers to the corresponding class, while “negative” refers to all other classes.

After determining the strategy for selecting the cut-off threshold, we calculate accuracy, sensitivity, and specificity values, which are shown in Table 3. Regarding accuracy, our method performs best for six classes (DR, RB, ERM, AMD, GS, and Normal) and second best for RVO. Remarkably, in terms of sensitivity, our method outperforms all reference methods for all classes. For specificity, our method achieves the best results for five classes (DR, ERM, AMD, GS, Normal) and comes in second for the other two (RB, RVO). It is worth noting that accuracy values are close to those of specificity. For each disease, sensitivity measures the performance in identifying positive samples, while specificity evaluates the identification of negative samples. Accuracy represents the performance in identifying both positive and negative samples. As the number of negative samples dominates the number of positive samples, accuracy values are closer to specificity than sensitivity. For a better understanding of the reported performance, we show the confusion matrix of our method in Figure 8. This is the run achieving the highest AUC score among five runs.

In this study, the proposed method demonstrates a significant improvement over three reference methods across all micro-average metrics, including accuracy, sensitivity, and specificity as shown in Table 4. The proposed method achieves the highest accuracy among all methods, showing an improvement of approximately 2.00% over the best-performing reference method. Furthermore, it also obtains the highest sensitivity, with an improvement of around 4.11% compared to the most sensitive reference method. Lastly, our method excels in specificity, showcasing an increase of about 1.52% over the reference method with the highest specificity. These results clearly highlight that our proposed method significantly outshines the reference ones, providing a more reliable and effective solution for the UFI classification task.

#### 4.2.2. Ablation Study

Our method incorporates three CNN backbones for classifying various diseases, with each backbone exhibiting distinct capabilities for classifying specific types of diseases. Choosing an appropriate backbone significantly influences the final output. Moreover, the sizes of ROIs must be taken into account, as they play a critical role in determining the amount of information fed into the CNN backbone. In this section, we show our selection for these components.

In this study, we seek to identify the most effective CNNs for our proposed method. To achieve this, we conduct a comprehensive evaluation involving four well-known deep learning architectures: Xception, ResNet50, MobileNetV3, and EfficientNetB3. These architectures have shown remarkable performance in various computer vision tasks, making them strong candidates for our experiment. The results in Table 5 reveal that EfficientNetB3 outperforms the other networks for two groups,(DR, RB, and RVO) and (ERM and AMD), demonstrating its superior performance in classifying these diseases. On the other hand, Xception exhibits the best performance in classifying GS. Overall, the experiment underscores the importance of selecting the most suitable network architecture for a specific task, as the choice of the architecture can significantly impact the overall performance and effectiveness of the classification process. Furthermore, the results highlight the potential benefits of using a combination of different architectures tailored to the specific characteristics of each disease group, ultimately leading to more accurate and efficient classification of ocular diseases in UFI.

**Figure 9:**
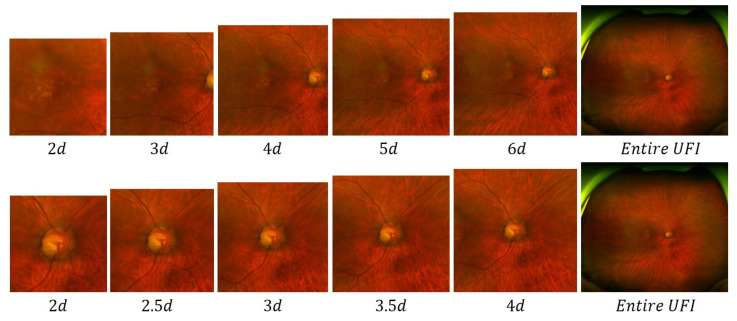
Illustration of ROIs with different edge lengths, *d* is the diagonal length of the detected optic disc box. The first and second rows show macula and optic disc areas, respectively.

In our study, we employ a method that extracts two ROIs within the UFI: the macula area and the optic disc area. The macula area is utilized for the detection of ERM and AMD, whereas the optic disc area is employed for the identification of GS. To optimize our method, we conduct experiments to determine the most suitable edge length for these two areas, which would result in the best classification performance. Our investigation involved varying the edge lengths of the macula and optic disc areas to identify the length that yields the highest AUC score. Visualizations with different lengths are shown in Figure 9. The results in Table 6 demonstrate that for the macula area, an edge length of 6 *d* leads to the best performance in detecting ERM and AMD. This optimal size ensures that the macula region is adequately captured, allowing the model to accurately identify the disease-specific features. For the optic disc area, the experiment revealed that the best edge length is 3.00 *d*. This size effectively covers the region surrounding the optic nerve head, which is crucial for detecting the subtle changes associated with GS. By optimizing the edge lengths of these two ROIs, our method can efficiently capture the most informative regions, leading to improved classification performance and ultimately contributing to more accurate and timely diagnoses of ocular diseases.

#### 4.2.3. Discussion

Our method becomes more effective due to two keys: focusing on the most important areas and reducing the output complexity of CNNs. Our method narrows down the regions of manifestation of each disease: the entire UFI for DR, RB, and RVO, the macular area for ERM and AMD, and the optic disc area for GS. By focusing solely on these regions, the classifiers not only examine the most relevant but also avoid the distraction from irrelevant parts. Furthermore, instead of having a single CNN trying to identify all six diseases simultaneously, which can be confusing and challenging, we divide this task among three separate CNNs. Each of these CNNs is responsible for fewer diseases, making their job more straightforward. In one paper [30], the authors reduce the number of outputs for each CNN using one binary classifier for each disease but this approach makes the dataset become more imbalanced, so, decreases the performance. The CNN #1 of our method is also a binary classifier but we achieve much higher performance than [30] because our CNN focuses on the most important part for diagnosing GS: the optic disc area.

**Figure 10:**
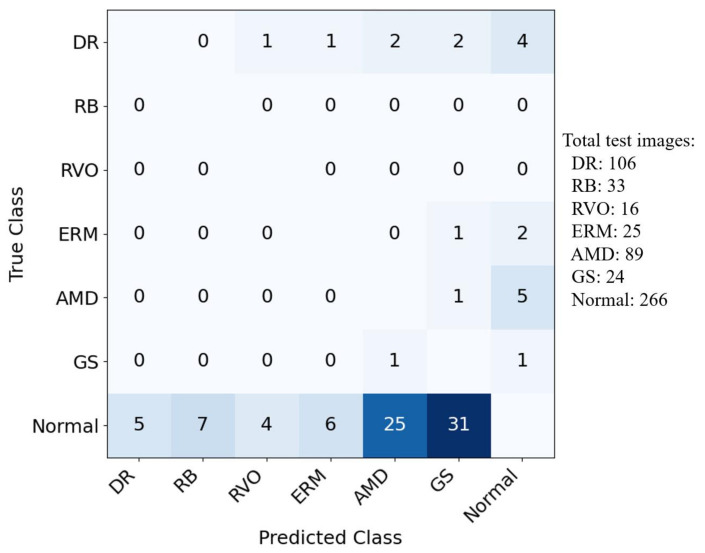
Number of misclassified images in each class.

To understand which classes are often confused with others, we analyzed the misclassification matrix, shown in Figure 10. For clarity, we only looked at images with a single label. We found that many normal images are wrongly predicted as AMD and GS. In UFI, there are often small yellow spots that look like drusen, a sign of AMD. This might be why normal images are mistaken for AMD, and vice versa. For GS, one sign is a thin layer at the back of the eye. However, some healthy young people might have this layer looking prominent, making the model think it is seeing early signs of glaucoma. Also, a larger ’cup’ in the nerve of the eye can be a sign of glaucoma. But some healthy people just naturally have a bigger cup. Telling the difference between a normal big cup and one due to disease can be hard. This could be why normal images are wrongly labeled as GS. Early signs of DR include tiny microaneurysms that the model might miss, leading it to label early DR images as normal. These ophthalmological findings pave the way for future improvements.

## 5. Conclusions and Limitations

In this study, we present a novel method for the multi-label classification of UFI, leveraging disease-specific knowledge to utilize the most informative regions for the accurate classification of individual ocular diseases. Our experiments on a dataset consisting of 5930 UFIs with six common diseases demonstrate the superior performance of the proposed method compared to existing works for the majority of classes and evaluation metrics, indicating its effectiveness in classifying ocular diseases. The results underscore the potential of our approach to provide valuable information to ophthalmologists, facilitating effective diagnosis and management of ocular diseases and ultimately contributing to improved patient outcomes. This innovative method showcases the benefits of integrating disease-specific knowledge into the classification process, paving the way for more advanced, accurate, and efficient diagnostic tools in the field of ophthalmology.

However, our study has its limitations. First, our dataset primarily encompasses six common ocular diseases, potentially limiting the generalization to other ocular conditions. Furthermore, the data-driven nature of our method means that its efficiency is closely tied to the quality and diversity of data available. Variations in imaging equipment or techniques could introduce inconsistencies that might affect performance. Another constraint is the inherent challenge of multi-label classification. There is always the possibility of overlapping symptoms between diseases, which might sometimes lead to ambiguous classifications. Despite these limitations, we believe our method holds immense promise and sets the stage for further research and refinement in the realm of UFI classification.

## Figures and Tables

**Table 1:** Comparison of different methods in terms of AUC score (%) for each class. Red and blue denote the best and second-best performance, respectively.

Method	DR	RB	RVO	ERM	AMD	GS
Lee et al. [30]	92.52 ± 0.80	97.63 ± 0.59	97.12 ± 0.61	90.58 ± 0.83	93.30 ± 0.54	88.93 ± 0.38
Wang et al. [31]	95.21 ± 0.23	98.55 ± 0.15	98.97 ± 0.15	91.16 ± 0.81	93.23 ± 0.20	89.14 ± 0.80
Zhang et al. [12]	94.66 ± 0.51	95.94 ± 0.96	98.46 ± 0.68	89.38 ± 0.38	91.14 ± 0.75	89.78 ± 1.12
Proposed	97.34 ± 0.25	99.14 ± 0.12	99.08 ± 0.13	96.21 ± 0.28	95.36 ± 0.10	95.07 ± 0.52

The results are reported with 95% confidence interval. DR: diabetic retinopathy, RB: retinal break, RVO: retinal vein occlusion, ERM: epiretinal membrane, AMD: age-related macular degeneration, GS: glaucoma suspect.

**Table 2:** Significance test result (*p*-value) for AUC scores. A value <0.05 indicates that the improvement of the proposed method over the referenced method is statistically significant.

	DR	RB	RVO	ERM	AMD	GS
vs. Lee et al. [30]	<0.05	<0.05	<0.05	<0.05	<0.05	<0.05
vs. Wang et al. [31]	<0.05	<0.05	0.31	<0.05	<0.05	<0.05
vs. Zhang et al. [12]	<0.05	<0.05	0.15	<0.05	<0.05	<0.05

**Table 3:** Comparison of different methods in terms of accuracy, sensitivity, and specificity. An underlined value denotes p<0.05 which means our improvement is statistically significant than the reference method. Red and blue denote the best and second-best performance, respectively.

Metric	Method	DR	RB	RVO	ERM	AMD	GS	Normal
	Lee et al. [30]	89.15 ± 1.63	93.26 ± 1.64	92.79 ± 1.97	87.31 ± 3.47	86.48 ± 3.64	87.38 ± 2.71	84.64 ± 0.51
Accuracy	Wang et al. [31]	91.29 ± 1.12	95.76 ± 0.35	97.26 ± 1.73	90.55 ± 1.17	86.74 ± 2.58	77.50 ± 2.98	85.57 ± 1.82
	Zhang et al. [12]	89.82 ± 1.68	92.32 ± 2.58	95.63 ± 0.87	81.20 ± 5.21	85.08 ± 3.44	85.64 ± 1.11	84.47 ± 1.47
	Proposed	93.02 ± 1.58	95.79 ± 2.17	95.76 ± 2.36	92.09 ± 1.97	87.58 ± 1.40	88.11 ± 1.90	87.15 ± 1.04
	Lee et al. [30]	79.85 ± 3.50	94.42 ± 3.09	95.24 ± 0.00	81.70 ± 4.08	87.77 ± 4.10	81.88 ± 1.23	75.52 ± 1.48
Sensitivity	Wang et al. [31]	86.67 ± 1.55	95.35 ± 0.00	95.24 ± 2.95	79.15 ± 3.59	87.20 ± 3.41	91.88 ± 3.68	73.06 ± 4.52
	Zhang et al. [12]	86.67 ± 2.97	90.70 ± 0.00	95.24 ± 2.95	82.98 ± 6.98	82.72 ± 4.23	86.25 ± 1.50	72.99 ± 3.47
	Proposed	91.63 ± 2.06	97.21 ± 2.66	96.19 ± 3.49	91.49 ± 3.73	90.87 ± 1.66	92.50 ± 3.13	76.27 ± 2.30
	Lee et al. [30]	91.70 ± 2.52	93.16 ± 1.99	92.70 ± 2.04	87.79 ± 4.09	86.21 ± 5.23	87.69 ± 2.92	92.03 ± 0.98
Specificity	Wang et al. [31]	92.55 ± 1.81	95.79 ± 0.38	97.34 ± 1.89	91.52 ± 1.54	86.77 ± 3.83	76.69 ± 3.34	95.71 ± 1.07
	Zhang et al. [12]	90.68 ± 2.92	92.45 ± 2.78	95.64 ± 0.95	81.05 ± 6.25	85.56 ± 4.87	85.61 ± 1.24	93.78 ± 0.76
	Proposed	93.40 ± 2.37	95.68 ± 2.47	95.74 ± 2.56	92.14 ± 2.33	86.90 ± 2.03	87.87 ± 2.13	95.95 ± 0.83

The results are reported with 95% confidence interval. DR: diabetic retinopathy, RB: retinal break, RVO: retinal vein occlusion, ERM: epiretinal membrane, AMD: age-related macular degeneration, GS: glaucoma suspect.

**Table 4:** Comparison by micro-average metrics (p<0.05). Red and blue denote the best and the second-best values, respectively.

Method	Micro-Average		
	**Accuracy**	**Sensitivity**	**Specificity**
Lee et al. [30]	88.83 ± 0.87	80.90 ± 0.68	90.27 ± 0.95
Wang et al. [31]	89.24 ± 0.85	81.55 ± 1.30	90.63 ± 0.86
Zhang et al. [12]	87.85 ± 1.05	79.41 ± 1.96	89.37 ± 1.26
Proposed	91.24 ± 0.74	85.66 ± 0.83	92.15 ± 0.61

The results are reported with 95% confidence interval.

**Table 5:** AUC scores with different CNN backbones. The backbone with the highest average value for each group is selected. Red value denotes the best result.

CNN Backbone	DR	RB	RVO	Average	ERM	AMD	Average	GS
MobileNetV3	95.90 ± 0.36	98.54 ± 0.20	99.43 ± 0.18	97.96	95.26 ± 0.41	94.14 ± 0.57	94.70	90.58 ± 0.63
EfficientNetB3	97.34 ± 0.25	99.14 ± 0.12	99.08 ± 0.13	98.52	96.79 ± 0.44	95.21 ± 0.29	96.00	93.03 ± 0.64
Xception	96.77 ± 0.21	98.86 ± 0.50	99.42 ± 0.29	98.35	96.10 ± 0.49	95.52 ± 0.06	95.81	95.07 ± 0.52
Resnet50	94.68 ± 0.95	98.38 ± 0.58	98.80 ± 0.49	97.29	95.75 ± 0.50	95.65 ± 0.57	95.70	90.03 ± 0.26

The results are reported with 95% confidence interval. DR: diabetic retinopathy, RB: retinal break, RVO: retinal vein occlusion, ERM: epiretinal membrane, AMD: age-related macular degeneration, GS: glaucoma suspect.

**Table 6:** AUC scores when varying the edge length of the ROIs. For the macula area, the size with the highest average value is selected.

(a) Macula Area			
**Edge Length** **of Macula Area**	**ERM**	**AMD**	**Average**
2 *d*	96.40 ± 0.31	94.52 ± 0.26	95.46
3 *d*	96.09 ± 0.50	95.15 ± 0.27	95.62
4 *d*	96.09 ± 0.44	95.40 ± 0.36	95.75
5 *d*	96.17 ± 0.30	95.28 ± 0.35	95.73
6 *d*	96.79 ± 0.44	95.21 ± 0.29	96.00
Entire UFI	93.96 ± 0.68	94.54 ± 0.30	94.25
**(b) Optic disc area**			
**Edge Length** **of Optic Disc Area**	**GS**		
2.00 *d*	92.55 ± 0.59		
2.50 *d*	94.32 ± 0.54		
3.00 *d*	95.07 ± 0.52		
3.50 *d*	94.89 ± 0.61		
4.00 *d*	94.81 ± 0.78		
Entire UFI	88.86 ± 0.49		

The results are reported with 95% confidence interval. ERM: epiretinal membrane, AMD: age-related macular degeneration, GS: glaucoma suspect.

## Data Availability

Data available on request (The data used in the study may be available depending on the corresponding author and/or IRB’s decision).
